# HIV-associated malignancies in Tunisia: a 30-year retrospective cohort study from a North African perspective

**DOI:** 10.1186/s13027-026-00779-y

**Published:** 2026-07-14

**Authors:** Sarra Yacoub, Manel Ben Selma, Souha Ghachem, Mariem Ben Ticha, Hela Knani, Mariem Garma, Taghrid Tlili, Safouene Frini, Badreddine Sriha, Wissem Hachfi

**Affiliations:** 1 Department of Pathology and Clinical Research and development Unit of pathology department, Farhat Hached University Hospital, Sousse, Tunisia; 2 Department of Infectious Diseases, Farhat Hached University Hospital, Sousse, Tunisia; 3 Private Family Medicine Practice, Sousse, Tunisia; 4 Faculty of Medicine of Sousse, University of Sousse, Sousse, Tunisia

**Keywords:** HIV, AIDS-defining cancers, Non-AIDS-defining cancers, Kaposi sarcoma, Non-hodgkin lymphoma, Tunisia, Africa

## Abstract

**Background:**

Despite major advances in antiretroviral therapy (ART), HIV-associated malignancies remain a significant cause of morbidity and mortality in low- and middle-income countries. In Tunisia, data on AIDS-defining cancers (ADCs) and non–AIDS-defining cancers (NADCs) among people living with HIV (PLWH) remain scarce.

**Methods:**

We conducted a descriptive retrospective cohort study over a 30-year period (1994–2024) at Farhat Hached University Hospital, Sousse, Tunisia. All PLWH who developed at least one malignant neoplasm were included. Epidemiological, clinical, immunovirological, pathological, and outcome data were retrospectively collected and analyzed.

**Results:**

Among 655 PLWH followed during the study period, 25 patients with a median age of 36 years developed malignancies, representing 3.8% of all PLWH. 27 malignancies were identified with 2 patients developing 2 distinct malignancies: a Kaposi sarcoma (KS) and a Non-AIDS-defining cancer (NADC). AIDS-defining cancers (ADC) predominated (81.5%), mainly KS (44.4%) and non-Hodgkin lymphoma (33.3%), while NADCs accounted for 18.5%. Cancer and *HIV diagnoses *were concomitant in 56% *of patients (n = 14)* and 92% of malignancies occurred within the first year following HIV diagnosis. Most patients presented with advanced immunodeficiency, with median CD4 counts at tumor diagnosis of 73 cells/mm³ for ADCs and 57 cells/mm³ for NADCs. Median overall survival was 1.9 years for ADCs and 3.7 years for NADCs.

**Conclusions:**

In this North African cohort, HIV-associated malignancies were predominantly AIDS-defining cancer and affected young adults with advanced immunosuppression, reflecting late HIV diagnosis for PLWH developing malignancies. Beyond strengthening early HIV testing and timely ART initiation, targeted patient education focusing on high-risk behaviors, along with efforts to reduce HIV-related stigma, are essential components to improve cancer prevention, early diagnosis, and outcomes among PLWH in resource-limited settings.

## Introduction

 Despite global progress in HIV control, the Middle East and North Africa (MENA) region remains one of the few regions where HIV incidence continues to rise [1]. Tunisia, a North African country with a low estimated HIV prevalence (0.1%), is not spared from this regional trend and has experienced a substantial increase in new HIV infections (40%) and HIV-related deaths (71%) over the past decade, reflecting ongoing transmission and gaps in HIV diagnosis [2].

HIV-associated malignancies remain a major cause of morbidity and mortality among people living with HIV (PLWH).With the widespread use of antiretroviral therapy (ART), life expectancy among PLWH has markedly improved, leading to a shift in the spectrum of HIV-associated malignancies. While the incidence of several AIDS-defining cancers (ADC) has declined in the ART era [3], non-AIDS-defining cancers (NADC) now account for an increasing proportion of cancer-related morbidity and mortality among PLWH [4]. This epidemiological transition has been well documented in Europe and in the USA [3, 5].

However, data from North Africa remain scarce [6, 7]. In Tunisia, the epidemiological and pathological characteristics of HIV-associated malignancies are still insufficiently documented. The few available studies have mainly focused on AIDS-defining cancers [6], and contemporary data encompassing both AIDS-defining and non-AIDS-defining malignancies among Tunisian PLWH remain limited.

Understanding cancer patterns in this population is essential to guide screening strategies, optimize management and ultimately improve outcomes for PLWH. This study aims to address this gap by investigating AIDS-defining and non-AIDS-defining malignancies among PLWH in Tunisia. The objectives were to describe the epidemiological and clinical characteristics of HIV infection in patients who developed cancer and to analyze the epidemiological, clinical, pathological and outcome characteristics of the different malignancies observed.

## Methods

### Study design and setting

This was a descriptive, retrospective cohort study conducted over a 30-year period (1994–2024) at the Infectious Diseases Department of Farhat Hached University Hospital, Sousse, Tunisia. The hospital is located in Sousse, a major coastal city in the eastern Sahel region of Tunisia. Sousse represents a secondary HIV referral center and serves a large surrounding catchment area, with a moderate HIV prevalence compared to the capital Tunis.

All medical records of PLWH followed during this period were reviewed, and every patient (with laboratory-confirmed HIV infection) who developed at least one malignant neoplasm — ADC (including Non-Hodgkin Lymphoma (NHL), Kaposi Sarcoma (KS) and cervical cancer) or NADC — was included in the analysis.

### Data collection

Data were collected retrospectively by reviewing patient medical records. Data collected included: age at HIV diagnosis, age at cancer diagnosis, sex, socioeconomic status, route of HIV transmission, HIV clinical and immunovirological data including Centers for Disease Control and Prevention staging (CDC clinical staging), CD4 cell count and plasma HIV viral load at enrolment and at tumor diagnosis;

Enrolment was defined as the first documented clinical evaluation in the Infectious Diseases Department of Farhat Hached University Hospital, at which immunological and virological data were available, regardless of whether HIV infection had been diagnosed previously elsewhere. Serological status of oncogenic viruses at enrolment, including hepatitis B virus (HBV), hepatitis C virus (HCV), Epstein–Barr virus (EBV). Pathological data were retrieved from pathological reports. Overall survival (OS) was defined as the time from the date of tumor diagnosis to the date of death from cancer, or to the date of last follow-up for patients who were alive at study closure (1 March 2025).

### Statistical analysis

Statistical analyses were performed using SPSS version 22.0 (IBM Corp., Armonk, NY, USA).

In patients who developed more than one primary tumor, each tumor was analyzed independently for descriptive, tumor-level analyses. Overall survival was estimated using the Kaplan–Meier method. Given the limited size of the NADC group (*n* = 4), no formal statistical comparison between the ADC and NADC groups was performed; all findings regarding this comparison are presented as descriptive only.

## Results

### Study population

Among 655 PLWH followed between 1994 and 2024, a total of 25 patients developed malignancies, representing 3.8% of all PLWH followed in our department during the study period. The study population was predominantly male (80%), with a male-to-female ratio of 4:1. The mean age at HIV diagnosis was 37.3 years, and the median age at cancer diagnosis was 36 years. Most patients belonged to a middle socioeconomic status (72%). Heterosexual intercourse was the predominant documented route of HIV transmission (*n* = 17; 68%), followed by men who have sex with men (*n* = 4; 16%).

### HIV related characteristics at diagnosis

**Fig. 1:**
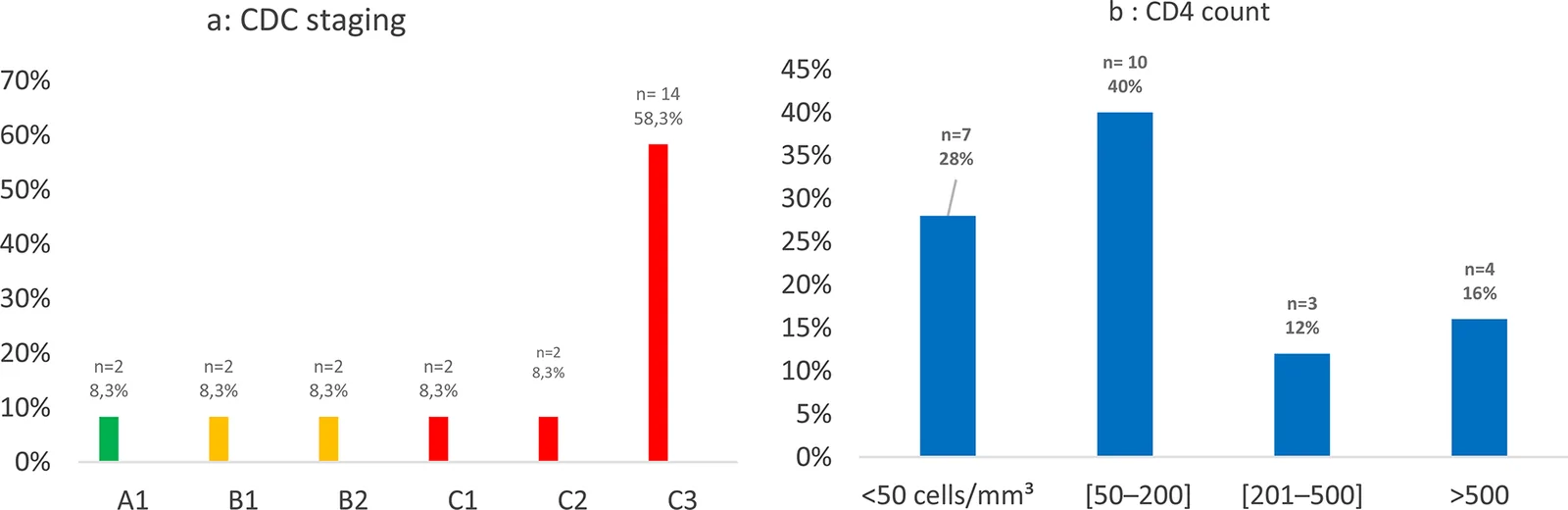
CDC staging (Fig. 1a) and CD4 count (Fig. 1b) among study patients (*n* = 24); Data unavailable for one patient (*n* = 24 for CDC staging and CD4 count); CDC STAGING: centers for disease control and prevention

HIV diagnosis was established under three main circumstances. In 36% of patients (*n* = 9), cancer was the initial manifestation leading to HIV detection. An infectious presentation prompted HIV diagnosis in 32% (*n* = 8). The remaining 32% (*n* = 8) were diagnosed under other circumstances, such as voluntary testing after high-risk behavior or blood donation screening. HIV infection was diagnosed at an advanced stage in most patients, with 75% patients classified as stage C, of whom 58.3% (*n* = 14) were classified as stage C3 according to CDC classification **(**Fig.1a**)**. The mean CD4 count at enrollment was 190 cells/mm³, with 68% of patients having CD4 counts below 200 cells/mm³ **(**Fig.1b**)**. The mean viral load was high (797,365 copies/mL).

Regarding co-infections, hepatitis B and C positivity were observed in 16% of patients each. EBV serology suggested past infection in 24% of tested patients, although testing was incomplete. HHV-8 serology was not routinely performed (Table 1).

**Table 1:** Oncogenic virus serology among study patients (*n* = 25)

Parameter	*n*	%
**Hepatits B (Serologic status)**		
Negative (susceptible)	17	68%
Positive serologic markers	4	16%
Resolved infection	2	8%
Immune (vaccination)	2	8%
**Hepatitis C (Serologic Status)**		
Negative	21	84%
Positive	4	16%
**EBV (Serologic Status)**		
Past infection	6	24%
Negative	9	36%
Not tested	10	40%

^a^ Data unavailable for one patient (*n* = 24 for CDC staging and CD4 count). CDC STAGING: Centers for Disease Control and Prevention; EBV: Epstein–Barr virus. HHV-8 serology was not performed at HIV diagnosis

### Tumor characteristics

**Fig. 2:**
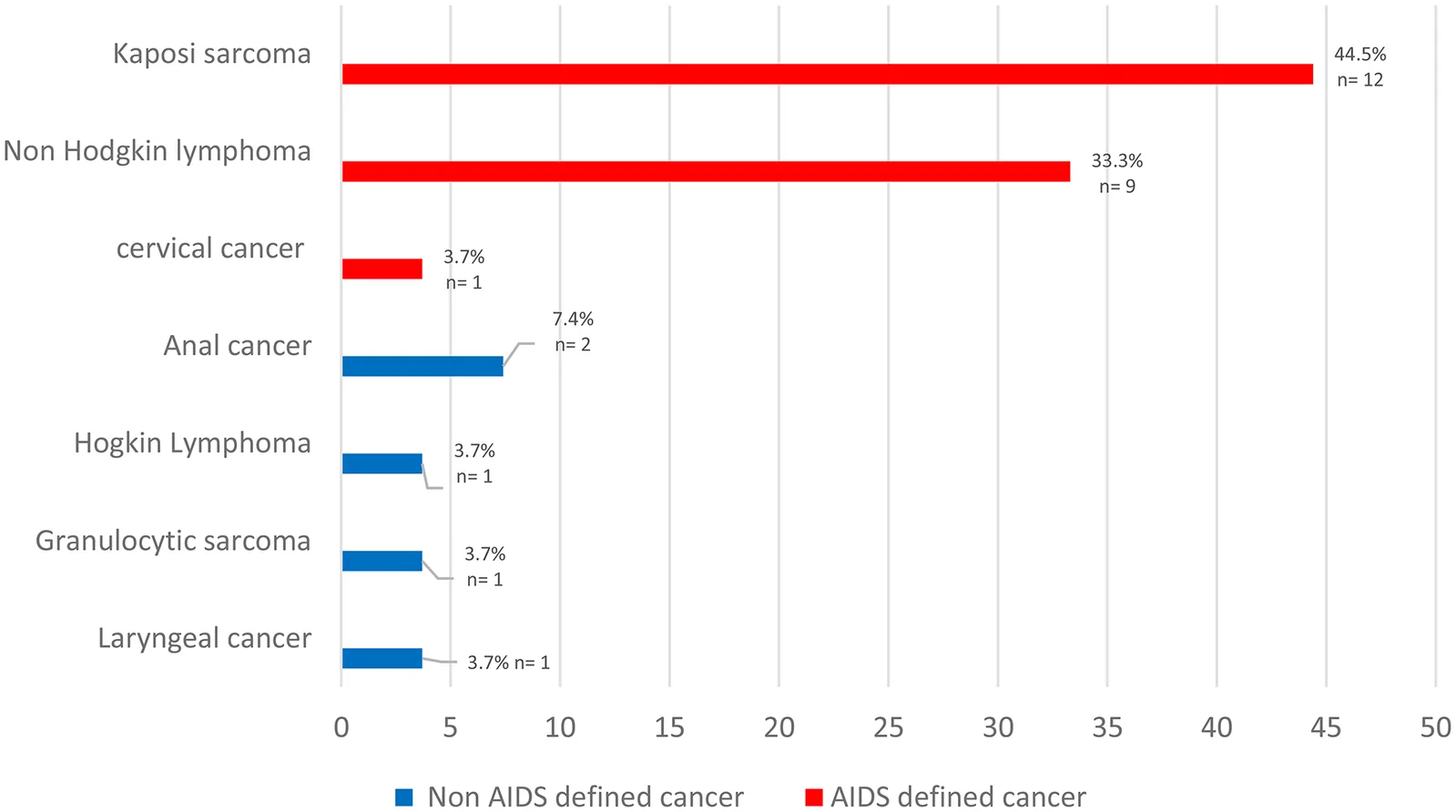
Distribution of tumor types by AIDS-defining status among the 27 malignancies. *Two patients developed 2 distinct malignancies

A total of 27 malignancies were identified among 25 patients;

2 patients developed 2 distinct malignancies (one patient developed Kaposi sarcoma (KS) then Hodgkin lymphoma; another developed KS then laryngeal cancer). Malignancy and HIV diagnoses were concomitant in 56% of patients (*n* = 14); cumulatively, 92% of cancers occurred within the first year of HIV diagnosis. ADC represented 81.5% of all tumors, while NADC accounted for 18.5%.The full distribution is presented in Fig. 2.

Compared to NADCs, ADCs were associated with lower CD4 counts at tumor diagnosis and higher viral loads, (Table 2). Given the small sample size of the NADC group (*n* = 4), no formal statistical comparison was performed.

**Table 2:** Epidemiological, biological and clinical parameters of ADC and NADC groups

Parameter	ADC (*n* = 21)	NADC (*n* = 4)
**Age**,** median [range]**,** years**	36 [27–57]	33 [30–65]
**Male sex**,** n (%)**	17 (81%)	4 (100%)
**Active smoker**,** n (%)**	13 (61.9%)	1 (25%)
**Alcohol use**,** n (%)**	11 (52.4%)	1 (25%)
**CD4 count at tumor diagnosis**, **median [range]**,** cells/mm**^**3**^	73 [4–598]	57 [7–83]
**Viral load at tumor diagnosis**, **median [range]**,** copies/mL**	380,000 [83–2,810,000]	34,000 [< 25–760,000]
**Adherent to ART**^**a**^, **n (%)**	14 (66.6%)	2 (50%)

^a^ ART: antiretroviral therapy. Given the limited size of the NADC group (*n* = 4), no formal statistical comparison was performed; data are presented as descriptive only

## AIDS-defining cancers (ADCs)

### Kaposi’s sarcoma

Twelve cases of KS were identified (44.4%). KS occurred predominantly in young males (*n* = 9; 75%) at a median age of 37.5 years. Patients were severely immunosuppressed at tumor diagnosis, with a median CD4 count of 49 cells/mm³ and a median viral load of 126,800 copies/mL. A disseminated form was documented in 58.3% of patients (*n* = 7). The most frequent anatomical pattern was combined cutaneous and mucosal involvement (41.7%; *n* = 5), followed by isolated cutaneous involvement (25%; *n* = 3), visceral involvement (16.7%; *n* = 2), and isolated mucosal or nodal location (8.3%; *n* = 1 each). Pathological diagnosis was confirmed by HHV-8 immunohistochemistry in all tested cases. Six patients (50%) died of KS-related disease, three achieved complete remission, and three remained under active treatment at study closure. The one-year survival rate was 58.3%, with no further decline observed through ten years.

### Non-hodgkin lymphoma

Nine cases of NHL were identified (33.3%). All patients were male, with a median age of 37 years. Patients presented with severe immunosuppression, with a median CD4 count of 96 cells/mm³ and a median viral load of 391,000 copies/mL.Extranodal involvement predominated (*n* = 5; 55.6%), with the gastrointestinal tract as the most frequent site (*n* = 3), followed by skin and head and neck locations. Advanced-stage disease was predominant, with 55.6% classified as Ann Arbor stage IVB. Diffuse Large B cell Lymphoma (DLBCL) was the most common subtype (*n* = 4; 44.5%), followed by plasmablastic lymphoma (*n* = 2; 22.2%), Burkitt lymphoma, ALK-negative anaplastic large cell lymphoma and extra-cavitary primary effusion Lymphoma (PEL) (*n* = 1 each; 11.1%). EBV was detected in 2 cases, 1 case of plasmablastic lymphoma and 1 case of extra-cavitary PEL. HHV-8 was only identified once in the extra-cavitary PEL case. Outcome was fatal in 88.9% (*n* = 8), with a median overall survival of 10 months. One patient with ALK-negative ALCL achieved complete remission.

### Cervical cancer

One case of invasive cervical cancer was identified (3.7%), occurring in a 36-year-old woman. At tumor diagnosis, CD4 count was 42 cells/mm³ and viral load was 702,000 copies/mL. The tumor was staged FIGO 3 C. Cervical biopsy revealed a poorly differentiated squamous cell carcinoma with associated high- and low-grade squamous intraepithelial lesions; HPV typing was not feasible. The patient was non-adherent to ART and received chemoradiotherapy in addition to ART. She died 40.5 months after tumor diagnosis.

### Non-AIDS-defining cancers (NADCs)

Five non-AIDS-defining cancers were identified in five male patients, comprising two anal cancers, one laryngeal cancer, one Hodgkin lymphoma and one granulocytic sarcoma. Clinical, virological, and outcome characteristics are summarized in Table 3.

ART adherence was poor in three of five patients. At last follow-up, four patients remained alive, with a notably marked immunodepression observed in 3 cases, prolonged survival observed in the laryngeal cancer case (21 years) and the granulocytic sarcoma case (18.9 years), despite the latter refusing ART.

**Table 3:** Clinical, virological and outcome characteristics of non-AIDS-defining cancer patients

Parameter	Anal cancer #1	Anal cancer #2	Laryngeal cancer	Hodgkin lymphoma	Granulocyticsarcoma
**Age (years)**	30	65	58	36	30
**Sex**	Male	Male	Male	Male	Male
**Histology**	Well-differentiated SCC	Well-differentiated SCC	Moderately differentiated SCC with sarcomatoid features	Nodular sclerosis HL	Granulocytic sarcoma
**Viral evidence**	HPV 35 (HR) + HPV 6 (LR)	Not tested	HPV negative	EBV positive	Not tested
CD4 at tumor diagnosis (cells/mm³)	7	57	NDᵃ	83	NDᵃ
Viral load at tumor diagnosis	760,000 copies/mL	Undetectable	Undetectable	34,000 copies/mL	NDᵃ
ART adherence	Adherent	Adherent	Non-adherent	Non-adherent	Refused ART
**Outcome / follow-up**	Alive at last follow-up (1.5 years)	Alive at last follow-up (1.5 years)	Alive (21 years)	Died (6 years after diagnosis)	Alive (18.9 years)

NDᵃ: not documented; SCC: squamous cell carcinoma; HL: Hodgkin lymphoma; HR: high-risk HPV; LR: low-risk HPV; ART: antiretroviral

### Outcome

**Fig. 3:**
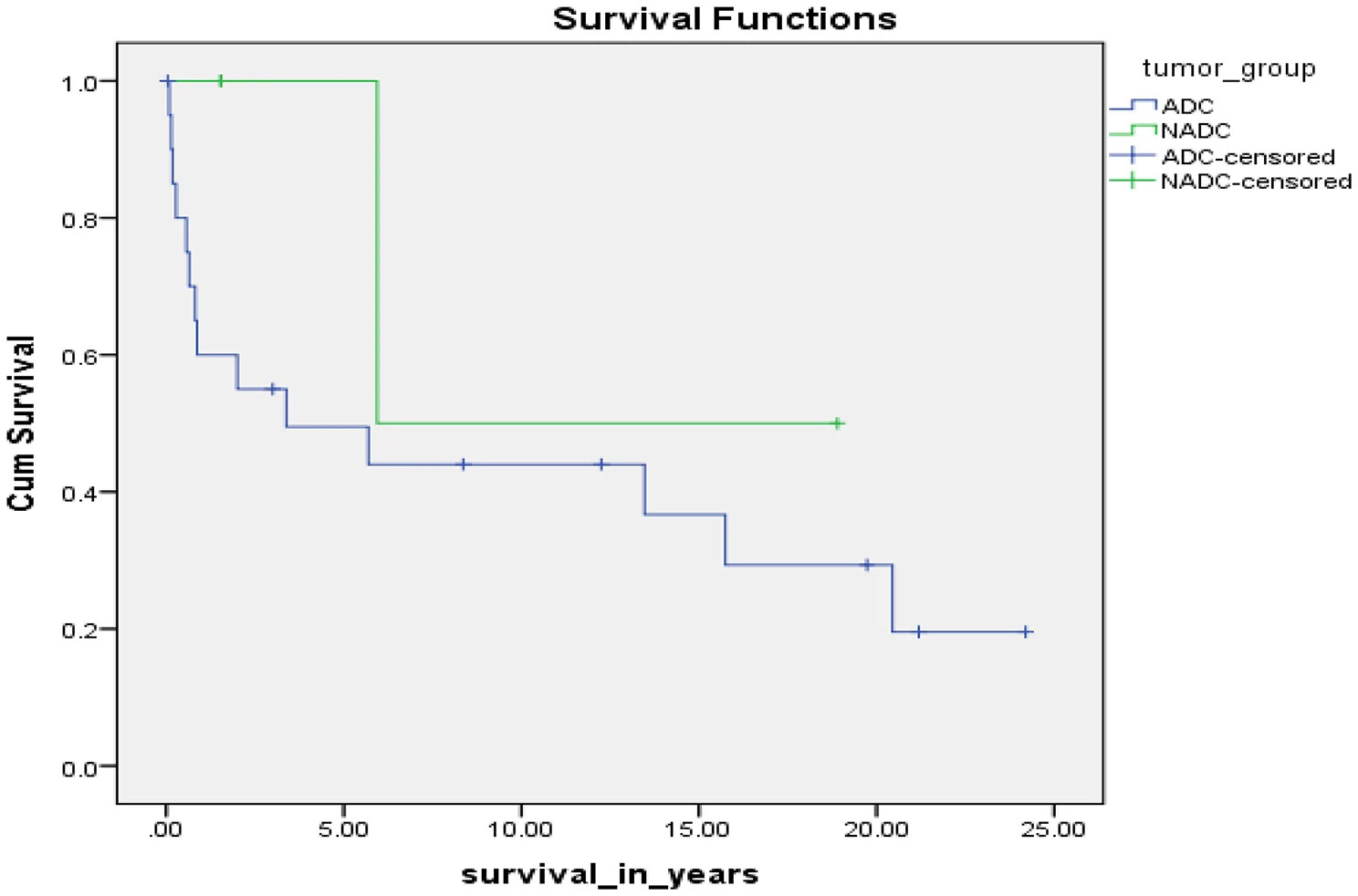
Kaplan–Meier survival curves comparing overall survival between AIDS Defining cancer (ADC) and Non-AIDS Defining Cancer(NADC) patients

Median overall survival was 1.9 years [0.04–24.2] in the ADC group and 3.7 years [1.5–18.9] in the NADC group. Figure 3 illustrates the Kaplan–Meier survival curves comparing overall survival between ADC and NADCgroups.

## Discussion

This study describes the clinical, pathological, and epidemiological characteristics of HIV-associated malignancies over a 30-year period in a Tunisian infectious disease department — one of the few longitudinal analyses of this kind in North Africa [7]. A cancer frequency of 3.8% was observed, lower than those reported in In the NA-ACCORD North American cohort, where cumulative cancer incidences of KS and NHL respectively reached 4.4% and 4.5% respectively by age 75 years among PLWH [8]. This difference, likely reflects younger patient age, diagnostic underreporting, competing mortality from opportunistic infections [9]. In Tunisia, data on HIV-associated malignancies remain scarce, and the absence of national cancer registries dedicated to PLWH, likely contributes to underestimating the true cancer burden in this population.

A marked male predominance was observed (80%), with a male-to-female ratio of 4:1, consistent with international cohorts where males represent over 70% of PLWH developing cancer [3];

### HIV clinical status and immunovirological parameters at enrolment

Late HIV presentation was a hallmark of this cohort: 75% of patients were at CDC stage C at enrolment, with 58.3% at the most advanced substage C3. This pattern mirrors findings from sub-Saharan African cohorts where 35–85% of patients present at WHO stage 3 or 4 [10]. Although HIV antiretroviral therapy (ART) is provided free of charge in Tunisia, diagnosis among PLWH with malignancies, continues to be established at advanced stages of infection. This pattern likely reflects limited risk awareness, social stigma, and the profound psychological burden associated with HIV disease among Tunisian and, more broadly, North African sociocultural context.

Regarding oncogenic viral co-infections, HBV co-infection was documented in 16% of patients, consistent with the upper range reported in Western cohorts (5–10%) [11]. EBV serology was positive in 24% of tested patients, though 40% were untested, limiting conclusions about its true oncogenic contribution in this cohort.

### ADC predominance: a reflection of persistent immunosuppression

AIDS-defining cancers accounted for 81.5% of tumors, a distribution strikingly different from the NADC-dominant patterns now observed in ART-experienced Western cohorts [12], and more similar to sub-Saharan African, where cancer frequently reveals late stages HIV [10]. The predominance of ADCs in our cohort likely reflects the young median age at diagnosis, combined with persistent severe immunosuppression. Studies have consistently shown that ADCs are strongly associated with profound immunodeficiency, while NADCs tend to emerge at older ages and after longer ART exposure with partial immune reconstitution [12]. A key observation of this study is that both ADC and NADC patients shared similarly low CD4 counts and elevated viral loads at tumor diagnosis. This convergence suggests that in the Tunisian context, cancer risk is primarily driven by late HIV diagnosis and suboptimal immune reconstitution even under ART treatment. This contrasts with high-income data showing that NADCs increasingly arise in virologically suppressed, immunologically reconstituted patients [13].

### Kaposi sarcoma and NHL: clinical and virological profiles

Kaposi sarcoma and non-Hodgkin lymphoma together accounted for 77.7% of all tumors, consistent with their established status as the leading HIV-associated malignancies in Africa and the Mediterranean basin [10, 14]. Both occurred predominantly in young males with profoundly low CD4 counts at tumordiagnosis, confirming that advanced immunosuppression remains the primary driver of ADC oncogenesis in this setting, even though more recent studieshighlighted that with ART intitation KS can occur with higher CD4 counts (≥ 350 cells/mm3) [15]. Histologically, HHV-8 immunohistochemistry confirmed the diagnosis in all KS cases, consistent with the established role of LANA-1 staining as a specific diagnostic marker [16]. Six patients (50%) died of KS-related disease, in line with outcomes reported in the ART era [17].

As for NHL, the DLBCL and plasmablastic lymphoma predominance (55.6%) align with global data on HIV-associated NHL subtypes [18]. According to Bibas et al., EBV involvement is detected in approximately 30% to 100% of HIV-associated non-Hodgkin lymphomas, depending on the histological subtype. The poor outcomes observed —88.9% NHL-related mortality with a 10-month median survival — reflect both disease aggressiveness and the challenges of oncologic care of such rare malignancies that are very specific to PLWH [19].

### Non-AIDS-defining cancers: heterogeneity and the role of oncogenic viruses

Despite representing only 18.5% of tumors, the NADC group displayed striking clinical diversity: anal squamous cell carcinoma with HPV co-infection, a laryngeal carcinoma with sarcomatoid transformation, Hodgkin lymphoma, and a granulocytic sarcoma. The detection of high-risk HPV genotype 35 in anal cancer is consistent with its increasing prevalence in African and immunocompromised populations [20]. Importantly, the finding that NADC patients also presented with profound immunosuppression (median CD4: 57 cells/mm³) challenges the assumption that NADCs in low-income settings arise predominantly in immunologically reconstituted patients — a pattern more typical of high-income cohorts [13].

### Survival and implications for clinical management

Median overall survival was 1.9 years in the ADC group and 3.7 years in the NADC group. These outcomes highlight the urgent need for integrated HIV-oncology care models adapted to resource-limited settings, including earlier ART initiationand cancer-specific multidisciplinary management and screening pathways.

### Limitations

This study has several limitations, including its retrospective, descriptive, single-center design, the small sample size—particularly in the NADC group (*n* = 4)—the absence of a control group, and the impossibility of formal statistical comparisons between groups due to the limited number of NADC cases. Despite these constraints, this work represents one of the few longitudinal characterizations of HIV-associated malignancies in North Africa over a three-decade period. It is based on a cohort of 655 people living with HIV followed over 30 years, among whom 25 developed malignancies at the Farhat Hached University Hospital, and provides valuable baseline data on AIDS-related and non–AIDS-related cancers from an under represented African settings.

## Conclusions

This 30-year retrospective study provides one of the few insights into HIV-associated malignancies in Tunisia and highlights the persistent predominance of AIDS-defining cancers, occurring at a young age and in the context of advanced immunosuppression. These findings underscore the ongoing impact of late HIV diagnosis despite the availability of antiretroviral therapy. Improving adherence to ART, is essential to reduce cancer-related morbidity and mortality in this population. In parallel, raising awareness about high-risk behaviours and actively combating HIV-related stigma must be among critical public health priorities to improve prevention, timely diagnosis, and outcomes among PLWH.

## Data Availability

No datasets were generated or analysed during the current study.
